# Mind the gap! Interdisciplinary approach to anterior chest wall reconstruction after total sternectomy

**DOI:** 10.1186/s13019-024-02743-6

**Published:** 2024-04-30

**Authors:** Olaf Michael Glueck, Denis Ehrl, Rudolf A. Hatz, Jan M. Fertmann

**Affiliations:** 1 Division of Thoracic Surgery, LMU University Hospital, LMU Munich, Marchioninistraße 15, 81377 Munich, Germany; 2 Division of Hand, Plastic and Aesthetic Surgery, LMU University Hospital, LMU Munich, Munich, Germany

**Keywords:** Sternectomy, Chest wall reconstruction, Chest wall stability, Reconstructive surgery

## Abstract

**Background:**

There are various reconstructive methods after total sternectomy. Reproducibility is scarce due to overall small patient numbers. Therefore we present a standardized, interdisciplinary approach for thoracic and plastic surgery.

**Methods:**

Four patients underwent interdisciplinary chest wall reconstruction with STRATOS® titanium bars and myocutaneous vastus lateralis muscle free flap in our center.

**Results:**

All patients reported chest wall stability after reconstruction. They reported good quality of life, no dyspnea, prolonged pain or impairment in lung function from rigid reconstruction. FEV1/FVC was overall better after surgery. Secondary wound healing was not impaired and there was no implant defect in follow up.

**Conclusions:**

We recommend an interdisciplinary surgical approach in chest wall reconstruction after total sternectomy. The combination of rigid reconstruction with titanium bars and a myocutaneous vastus lateralis muscle free flap renders excellent results in patient satisfaction and is objectifiable via spirometry.

## Background

Total sternectomy is rare but can be necessary due to oncological and non-oncologic disease. Most primary and secondary tumors of the anterior chest wall involve only small parts of the sternum, if at all. It is mainly connective tissue tumors like osteosarcoma or chondrosarcoma, or metastasis. Benign sternum destruction is a rare complication in 1–2% after open cardiac or aortic surgery. Small defects can develop to deep sternal wound infections (DSWI) with subsequent osteomyelitis [1, 2]. Failure of less-invasive treatment options leads to the need of total sternectomy and in the following, a robust and aesthetic reconstruction of the anterior chest wall. This is essential for stability, functionality and quality of life [3]. Reconstructive methods range from mesh – methylmethacrylate – mesh sandwiches or titanium mesh grafts to titanium bars and individualized 3D-printed sternum interponates and cadaveric sternal allografts, mostly described in case reports [4–9].

We present a small series of an interdisciplinary reconstructive surgical approach in four patients using titanium bars (STRATOS™, MedXpert GmbH, Eschbach, Germany) and free myocutaneous vastus lateralis muscle flap coverage.

## Patients and methods

### Ethical statement

All data presented was gathered after approval of the institutional Ethics Committee of the Ludwig-Maximilians University Hospital of Munich (reference no 21–0475). Patients have given consent for use of their images.

We present an interdisciplinary surgical approach in four comparable cases of anterior thoracic wall reconstruction after total sternectomy. Indications were either malignancy or deep sternal wound infection (see Table 1). All patients were male, the mean age was 62,5 years (53–72) at time of surgery. Patient A, B and D underwent various treatments for sternal defect after full or sternectomy before and all patients presented with different sets of comorbidities (Table 1). We performed computed tomography (CT) angiography in all patients for evaluation of target vessels for anastomosis and for thorough surgical planning. All patients underwent spirometric testing before and after surgery. For comparability the FEV1/FVC ratio was used to describe changes in lung function. Furthermore, subjective postoperative breathing mechanics were evaluated by observation and questioning the patients before and after surgery.

**Table 1:** Demographical data, follow-up and pulmonary function of the selected patients

	Patient A	Patient B	Patient C	Patient D
Age (y)	60	72	65	53
Sex	m	m	m	m
BMI (kg/m²)	33.2	27.2	26.7	35.0
Cause of defect	aortic arch replacement	triple bypass	NSCLC	aortic valve replacement traffic accident
Sternal defect	DSWI	DSWI	sternum metastasis	DSWI
Pretreatment	sternal rewiring omental interpositioning	omental interpositioning	left upper lobe lobectomy chest wall resection	sternal rewiring sternal osteosynthesis
Comorbidities	arterial hypertension OSAS	atrial fibrillation depression renal replacement therapy IDDM	NSCLC renal insufficiency	OSAS hepatic steatosis IDDM
Revision surgery	none	secondary wound closure	none	STRATOS bar replacement
ICU stay (d)	2	1	8	14
Hospital stay (d) overall vs. post surgery	10/9	22/14	42/29	25/24
Follow-up	47 m, alive	45 m, alive	10 m, dead	3 m, alive
Tiffeneau-index pre to post surgery	+5% (85 - 87%)	-5% (69% - 64%)	+11% (64 - 75%)	+/- 0 (83%)

Abbreviations: OSAS - obstructive sleep apnea syndrome; IDDM - insulin dependent diabetes mellitus; NSCLC - Non small-cell lung cancer

For patients A to C we advised wound debridement and negative pressure wound therapy (NPWT, V.A.C.® System, 3 M + KCI, St. Paul, MN, USA) for defect sanitation before surgery. The indication for surgery was given if the defect was considered rehabilitated with negative microbiological swabs and clean wound bed alongside declining infectious parameters in laboratory tests. In surgery, the destroyed sternum and adjacent ribs were resected with thorough debridement of the adherent structures, followed by reconstruction with four titanium bars and defect coverage with myocutaneous musculus vastus lateralis (MVL) free flap. Free MVL flap was chosen after failure of less invasive coverage attempts, for example pectoralis major suture. Furthermore, since the defects had to be conditioned beforehand, there was scar tissue build-up along the sides after NPWT, resulting in difficult mobilization of other graft options like latissimus dorsi grafts or pectoralis major muscle flaps. All patients were distinctly obese, resulting in additional tension on the anterior chest wall in supine position, therefore a free MVL flap was chosen to reduce tension and ensure better wound healing.

**Fig. 1:**
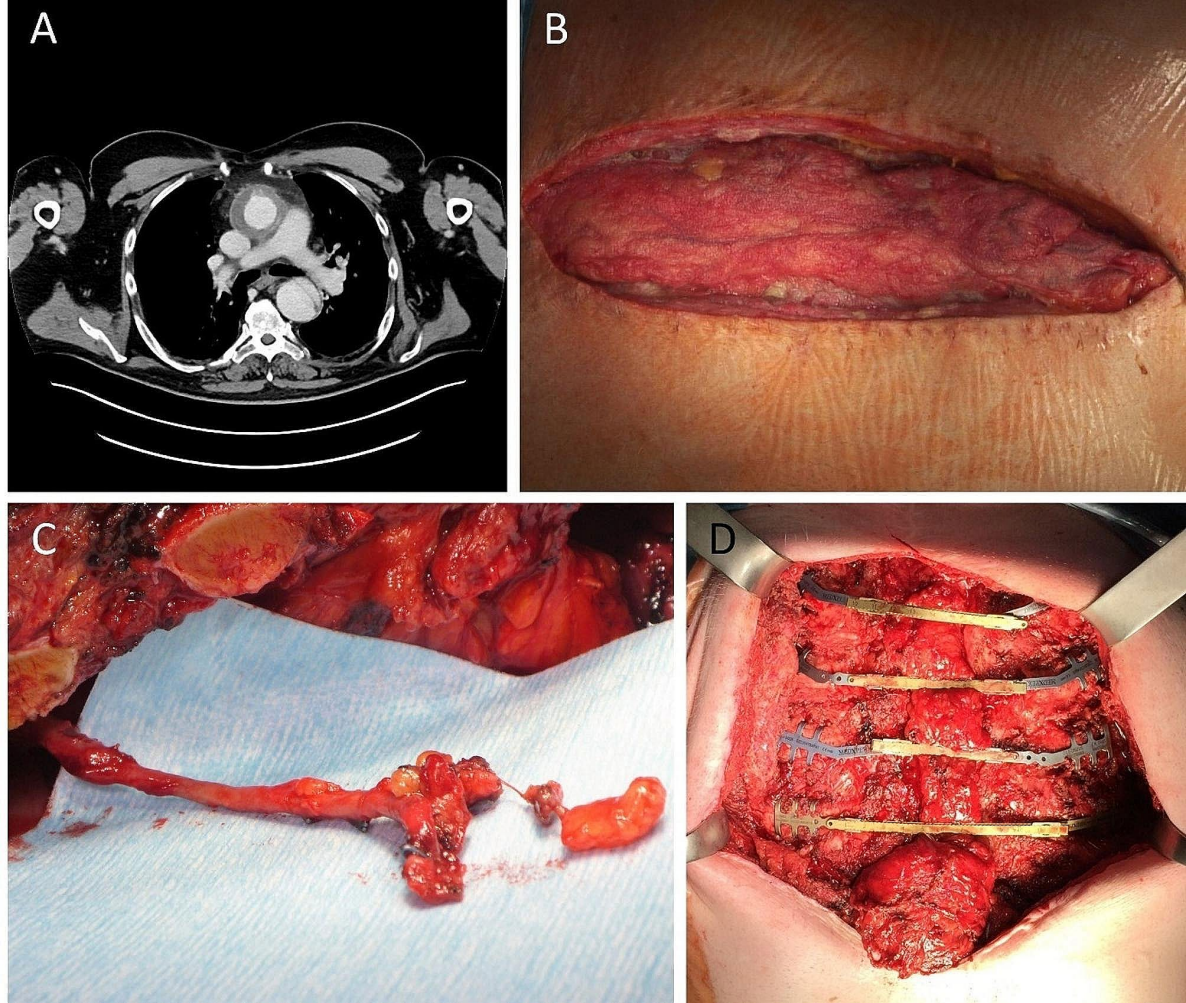
(**A**) computed tomography transversal, note missing sternum; (**B**) sternectomy wound after preconditioning with NPWT, note interpositioned omentum flap; (**C**) internal mammary artery preparation for vessel anastomosis; (**D**) rigid stabilization with alternating clip implantation

Sternal defect preparation: Patients were placed in supine position. The sternal wound defect was widely resected, in the oncological case to maximum extent to achieve tumor-free margins. For DSWI, all necrotic tissue was resected up to healthy bone material. For vascular anastomosis of the myocutaneous flap, the right internal mammary artery or the inferior thyroid vessels were carefully prepared and blocked with heparin-sodium solution (Fig. 1C). In case of abdomino-mediastinal herniation after previous omentum interpositioning, the defect was repaired using IPOM mesh graft technique or direct suture after repositioning the omentum to intraabdominal.

Defect coverage: Ribs 2–5 were parasternally freed from connective and muscle tissue for clip placement. Clips with alternating rigid and flexible connectors were placed. The connecting bars were then attached to the clips in cranial to caudal order from the side after thorough measurement and corresponding shortening. (Fig. 1D).

Free flap preparation: Following rigid chest wall reconstruction, myocutaneous vastus lateralis muscle (MVL) free flaps were raised and micro-anastomosed to the vessels mentioned above. The left or right vastus lateralis was chosen as donor site and the planned flap measured to fit the sternal defect size. It was then thoroughly dissected and raised with a long cranial pedicle for re-anastomosis. The respective donor site wound was primarily closed by suture in all patients.

Free flap replantation: The raised MVL flap was then directly re-anastomosed on the prefabricated vessels (as mentioned above) with running suture, after shortening the vessels appropriately to avoid kinking. Flap blood flow was continuously monitored by duplex ultrasound during surgery. Post surgery, blood flow monitoring was gradually reduced over the course of 10 days. For postoperative thrombosis prophylaxis, the patients received iv heparin or, in case of HIT, argatroban. Drainages were placed in all levels (pleural, mediastinal, sub-flap), if necessary.

All patients received perioperative antibiotic prophylaxis with cefuroxime or in case of known allergy clindamycin, followed by broad-spectrum antibiotic (piperacillin/tazobactam) for a minimum of three to five days, or pathogen-appropriate treatment in case of positive swab.

**Fig. 2:**
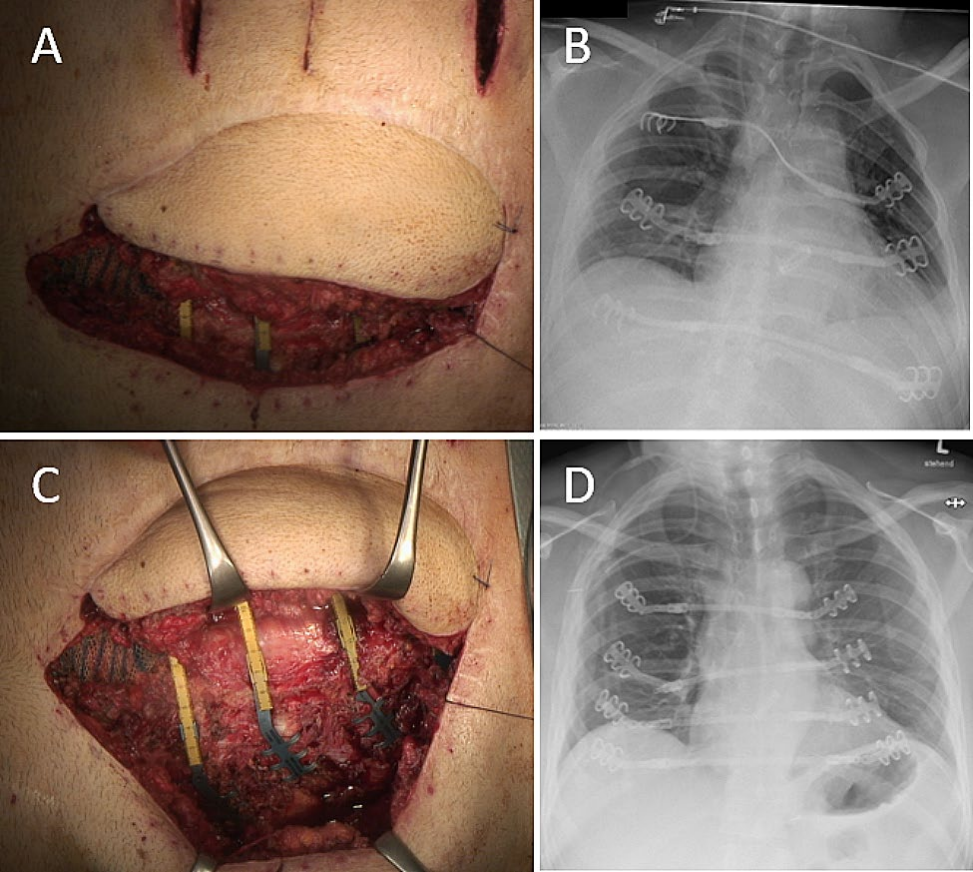
(**A**) Semi-elevated flap for bar placement, note: top of picture represents patients left side; (**B**) bar deformity after mobilization; (**C**) bar revision with semi-elevated flap, note: top of picture represents patients left side; (**D**) correct placement of STRATOS system after revision surgery

Patient D initially refused rigid reconstruction and underwent defect coverage with MVL free flap only. There was prolonged thoracic wall instability and the patient consented to secondary rigid reconstruction. The left half of the flap was lifted, and the left sided clips placed (Fig. 2A). The right-sided clips were placed through single incisions of the right chest wall and the connecting bars placed from left to right under the flap.

## Results

**Fig. 3:**
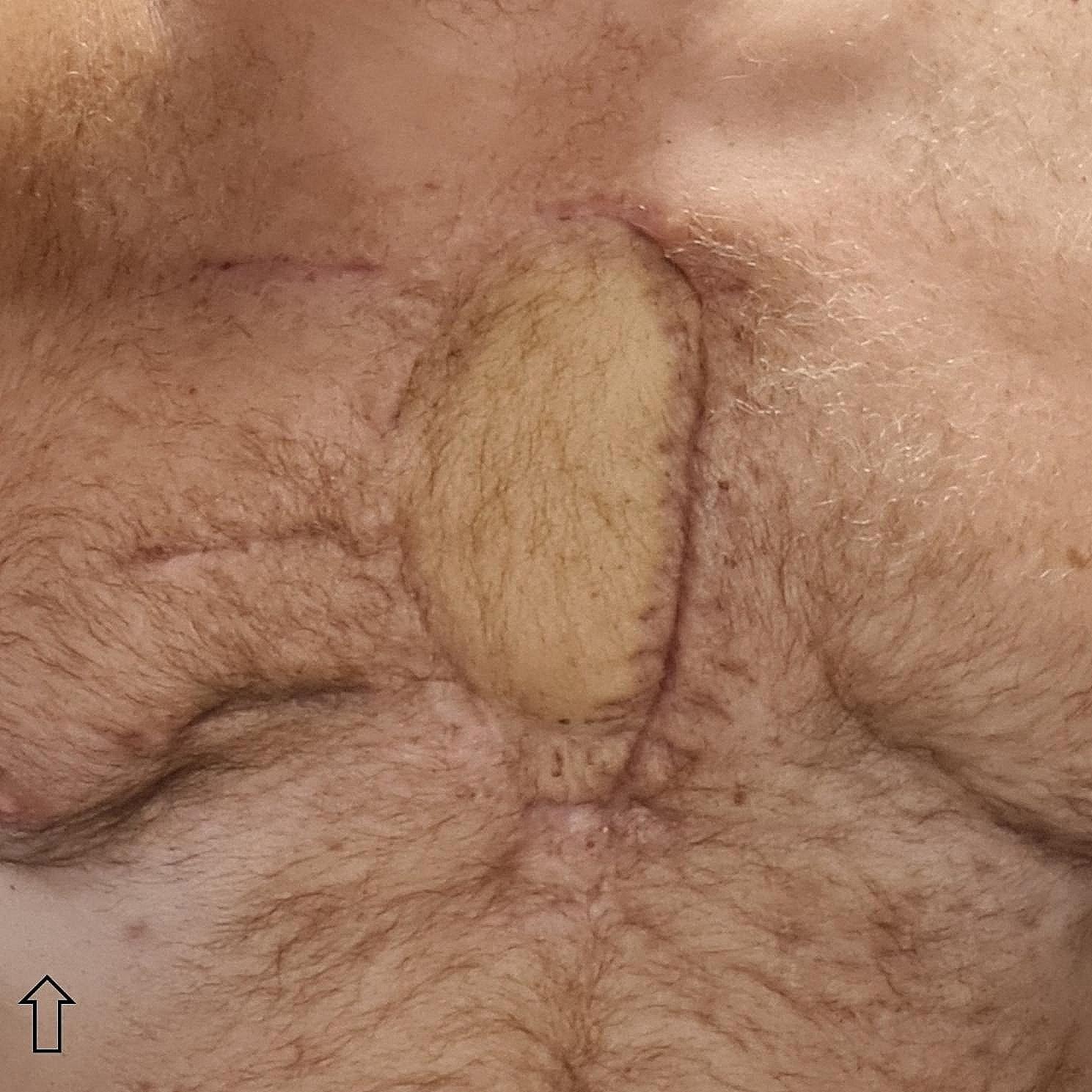
Fully healed MVL flap in patient D, two months post-surgery; *Note*: arrow indicating cranial direction

All patients were primarily extubated postoperatively, still in the operating theater. They reported good chest wall stability and no impairment of breathing mechanics, consistent with pre- and postoperative lung function testing. The FEV1/FVC ratio showed a mean increase of + 2.5% (see Table 1), while the focus was mainly on stable breathing mechanics after rigid reconstruction of the anterior chest wall. The mean defect size was 229,8 cm² (200–400 cm²). Postoperative hospital stay was 19 days (9–29), with a mean ICU stay of 6 days [1–14]. Postoperative pain syndrome was evaluated by visual analog scale (VAS) and managed through peridural anaesthesia in two cases, in combination with WHO standard by non-steroidal antiphlogistics alongside oral or intravenous opioid drugs. Control chest x-ray imaging confirmed stable clip and connection bar placement. The patients furthermore reported excellent aesthetic outcome with improved quality of life and excellent myocutaneous flap vitality. There were no secondary complications to the flap or at the clip-bone interface even after radiation therapy in one patient. There was no impairment in function or any other complication observed at the flap donor site. At d3 after surgery in Patient D, chest x-ray revealed deformed titanium bars from excessive mobilization, requiring revision surgery (Fig. 2B). The MVL flap was semi-lifted in a protective manner and remained vital (Fig. 2A, C). This patient eventually returned to his profession as a singer without impaired breathing mechanics and good lung function. The aesthetic outcome is presented in Fig. 3. Mean follow-up was 26 months (2–47), with one patient lost-to-follow up and one patient deceased.

## Discussion

The main limitation of this report is the small case number, but we can conclude that large-scale reconstructive surgery of the anterior chest wall after total sternectomy can be performed without negative impact to overall outcome and rare minor complications. We consider the main indication for the presented procedure to be need for complete sternal resection with failure of less invasive reconstructive procedures. As contraindications, we identified ongoing wound infection, no target vessel for flap anastomosis and lack of sufficient bone tissue (osteomyelitis) for secure clip placement. Short intensive care unit and hospital stay as well as improved breathing mechanics result in overall improved quality of life in this patient cohort. This is concordant with previously published case reports which used different reconstructive approaches for sternum defects [3–7, 10, 11]. While many reports are individual treatment approaches with individualized reconstruction methods, we are relying on a commercially available system (STRATOS®) to facilitate reproducibility. Similar techniques have previously been described in works of Berthet et al. [12, 13]. However, it should be emphasized that in the previous reports there were three cases of total sternectomy, which showed smaller defects that could be covered without free myocutaneous flap. Also, we cannot agree with reports of bar fractures [12, 14], but in contrary report on bar stability even after severe deformation in patient D. This may in part be due to usage of rotating connector parts in this cohort, ensuring more flexibility with mobilization and breathing mechanics.

## Conclusion

Thorough planning is indispensable for solid and aesthetic outcome in large reconstructive surgery. We demonstrate the feasibility of a combined rigid and aesthetic soft tissue reconstruction of the anterior chest wall after complete sternectomy with reestablished breathing mechanics and the possibility to qualify oncologic patients to further therapy in selected cases. In combination with growing experience in large-extent reconstructive surgery flap interposition, we are gaining confidence in complex cases of sternum reconstruction.

## Data Availability

The data used and/or analysed during the current study are available from the corresponding author on reasonable request.

## Abbreviations

DSWI: Deep sternal wound infection
STRATOS: Strasbourg Thoracic Osteosynthesis System
FEV1: Forced exsipratory volume in 1 s
FVC: Forced vital capacity
NPWT: Negative pressure wound therapy
MVL: Myocutaneous vastus lateralis
